# A new dynamical layout algorithm for complex biochemical reaction networks

**DOI:** 10.1186/1471-2105-6-212

**Published:** 2005-08-26

**Authors:** Katja Wegner, Ursula Kummer

**Affiliations:** 1 Bioinformatics and Computational Biochemistry, EML Research, Schloss-Wolfsbrunnenweg 33, D-69118 Heidelberg, Germany

## Abstract

**Background:**

To study complex biochemical reaction networks in living cells researchers more and more rely on databases and computational methods. In order to facilitate computational approaches, visualisation techniques are highly important. Biochemical reaction networks, e.g. metabolic pathways are often depicted as graphs and these graphs should be drawn dynamically to provide flexibility in the context of different data. Conventional layout algorithms are not sufficient for every kind of pathway in biochemical research. This is mainly due to certain conventions to which biochemists/biologists are used to and which are not in accordance to conventional layout algorithms. A number of approaches has been developed to improve this situation. Some of these are used in the context of biochemical databases and make more or less use of the information in these databases to aid the layout process. However, visualisation becomes also more and more important in modelling and simulation tools which mostly do not offer additional connections to databases. Therefore, layout algorithms used in these tools have to work independently of any databases. In addition, all of the existing algorithms face some limitations with respect to the number of edge crossings when it comes to larger biochemical systems due to the interconnectivity of these. Last but not least, in some cases, biochemical conventions are not met properly.

**Results:**

Out of these reasons we have developed a new algorithm which tackles these problems by reducing the number of edge crossings in complex systems, taking further biological conventions into account to identify and visualise cycles. Furthermore the algorithm is independent from database information in order to be easily adopted in any application. It can also be tested as part of the SimWiz package (free to download for academic users at [1]).

**Conclusion:**

The new algorithm reduces the complexity of pathways, as well as edge crossings and edge length in the resulting graphical representation. It also considers existing and further biological conventions to create a drawing most biochemists are familiar with. A lot of examples can be found on [2].

## Background

With the development of sophisticated experimental technology scientists are trying to understand the huge cellular biochemical network of living cells in its entirety. The complexity of this ambitious goal requires the additional use of computers to be able to analyse the data resulting from high-throughput experiments. Computational approaches include the usage of modelling and simulation of biochemical processes which offers new insights into the way biochemical reactions interact with each other and new perspectives for drug development.

Modelling and simulating increasingly complex biochemical networks, however leads again to masses of data. In order to facilitate the understanding of the results, sophisticated visualisation techniques are required. Therefore, visualisation techniques for use in bioinformatics and computational biochemistry have attracted more and more attention in the last years.

One common example for such visualisations is the graphical representation of biochemical reaction networks/pathways. A graphical representation offers the advantage that the topology of the network which is tightly linked to its function, is easily depicted. This topology information is lost when a researcher is confronted with just a list of biochemical reactions. Of course, graphical representations exist that are hand-made and static (e.g. in biochemistry books).

Many examples of graphical representations on the computer are also static, e.g. KEGG [3]. Being static offers no flexibility in the level of detail or in the exact information depicted by the respective graphs. To get rid of this problem dynamic visualisation techniques arose that enable the user on the fly to see and change only those pathways that are needed at time of viewing [4].

In this paper we concentrate on a new dynamic layout algorithm for the graphical representation of complex reaction networks. In such a graph consisting of nodes and edges, the nodes of the graph represent the compounds and the edges the reactions between these compounds. The direction of an edge shows the direction of the reaction. If an edge points from *n*_1 _to *n*_2_, *n*_1 _is the substrate and *n*_2 _the product of the respective reaction.

Often there is a differentiation between two types of compounds, main and side compounds. Main compounds lie on the backbone of the pathway, e.g. in linear pathways they participate in adjacent reactions [5]. All other compounds in this pathway are considered as side compounds.

Dynamic graph layout algorithms try to visualise the graph in such a way that it is easy to survey. This means that crossing of edges is avoided as much as possible. Nodes and labels have to be placed such that they do not overlap. This task can in principle be performed by standard graph layout algorithms, however, there are reasons why this is not suffcient in the context of bioinformatics. One of these reasons is the very high degree of connectivity in complex biochemical networks and the other reason is that there is a certain way that biochemists are used to seeing these graphs that does not match the way a standard layout algorithm would represent a complex biochemical network (see [6] for a detailed discussion). Therefore, several specific dynamic layout algorithms for metabolic pathways have been developed in the past.

The algorithm by Karp et al. [5,7] uses a divide-and-conquer method (project BioCyc). In the first step the graph is decomposed into subgraphs. These subgraphs are drawn according to their topology (linear → hierarchical graph layout, cyclic → circular graph layout, branched → tree layout). In the second step a hierarchical layout algorithm assembles these subgraphs to a whole graph.

BioPath [6,8] is a dynamic electronic version of the *Boehringer Biochemical Pathway *map by Michal [9,10]. It uses an improved hierarchical layout algorithm [11]. However, BioPath is currently not available. As part of the PathDB project Mendes et al. (personal communication) developed a PathwayViewer that consists of an improved hierarchical [11] and an individual circular layout algorithm. Additionally, they allow the user to edit the final drawing.

The above approaches also make more or less use of additional information in specific underlying databases, e.g. information about the order of reactions, side compounds etc. This is an advantage when aiming at the best graphical representation of data in the database, e.g. metabolic pathways. However, it also often restricts the use of the layout algorithm to these applications. Simulation and modelling tools that also want to make use of sophisticated visualisation techniques cannot rely on additional data in most cases. A graph layout algorithm used in these tools has to work, e.g. on the basis of the information as presented in a SBML file [12]. SBML files only contain explicit data about the individual reactions present in a specific biochemical network. Furthermore, previous approaches first calculate the coordinates of the main compounds and subsequently place the side compounds separately as labels near the edge representing the reaction in which they take part (one single label for each occurrence).

In contrast Rojdestvenski [13] uses a modified spring-embedding layout algorithm [14] for 3D-representations mainly. The algorithm considers main and side compounds as nodes during the layout process but with different priorities. First only the main compounds are placed. Second the algorithm is started again with the main compounds and side compounds at the same time, but with frozen coordinates for the main compounds. In contrast to the other three projects the side compounds are treated as nodes of the graph and their coordinates are determined with the spring-embedder algorithm. Furthermore, each side compound occurs only once instead of one node for each occurrence in the network. However, this approach leads to many edge crossings in a graph with highly connected side compounds.

In 2001 Becker et al. [15] developed a divide-and-conquer method similar to Karp et al. Unlike Karp et al. they only differentiate between cyclic and hierarchical subgraphs in the graph and decompose the subgraphs with the help of a force-directed algorithm [16,17]. However, this method is only able to handle main compounds.

Nevertheless, we chose this algorithm as basis for our work, since cyclic and hierarchical structures are the two basic topologies in which every complex biochemical network/pathway can be separated. In addition, the Becker et al. algorithm is not linked to a specific database and is therefore easily adjusted to different environments and needs.

**Figure 1:**
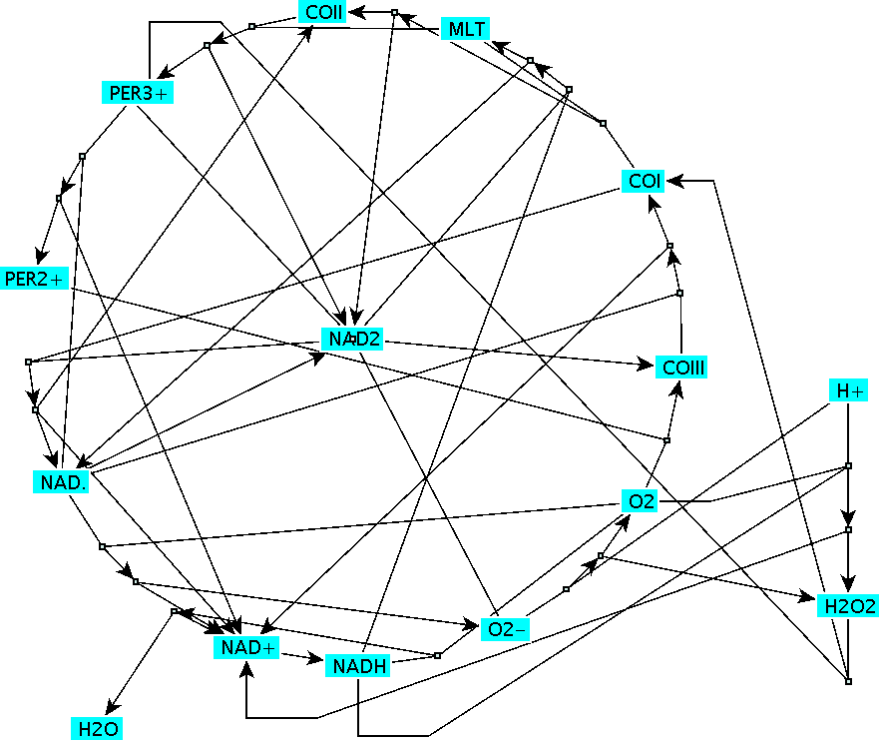
**Visualisation of the PO reaction [18]**. This picture is a result of the Becker et al. algorithm. The seventeen reactions of this pathway are hardly recognisable.

**Figure 2:**
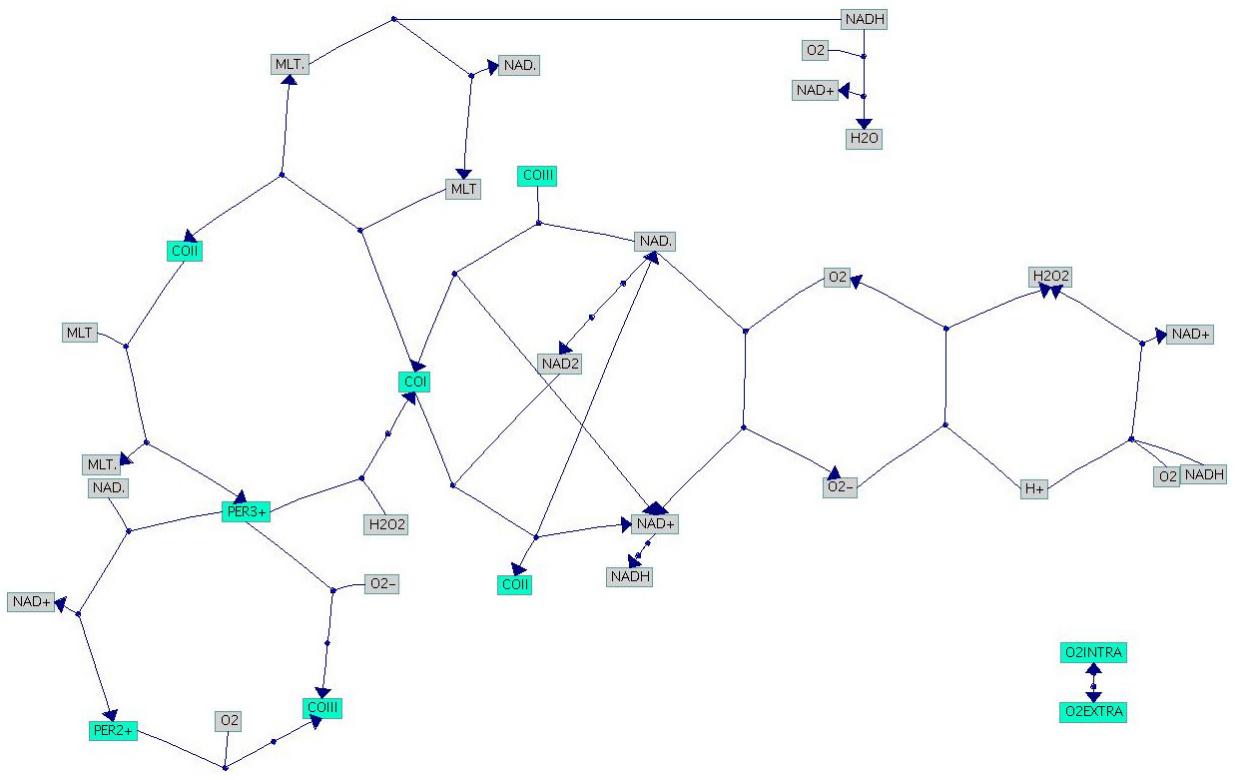
**Visualisation of the PO reaction [18]**. The picture is a result of our new layout algorithm and shows the reactions in a much clearer way compared to Fig. 1.

All of the above algorithms work reasonably well for small to medium sized networks. However, the complexity of studied biochemical networks is increasing. For large and complex pathways the existing pathway layout algorithms often face a problem with respect to the number of edge crossings. Such complex pathways contain highly connected nodes and cycles that share nodes with other cycles. In addition, biological conventions stress the importance of cycles, even small cycles in general since such structures represent important recycling processes and shortcuts in the system. However, existing algorithms do not take these conventions into account. We tested e.g. the Becker et al. algorithm with seventeen elementary reactions of the Peroxidase-Oxidase reaction (PO-reaction [18]). These elementary reactions interact strongly with each other and two recycling processes are present. The Becker et al. algorithm created a tangle of nodes and edges which is impossible to survey (Figure 1, as comparison Figure 2 shows the same reactions drawn by our new algorithm). In the following sections we show how the new algorithm solves these problems, reduces edge crossings and presents the reaction in a way that corresponds more closely with biological conventions. This is achieved by identifying even small cycles and by splitting nodes to improve the readability of the dynamic drawing.

## Results

As the starting point for our algorithm we used the implementation of the Becker et al. algorithm that is based on the Java graph library YFiles [19]. In general, our algorithm differs from the Becker et al. algorithm in the following ways: it is able to join and split nodes and to detect smallest cycles or cycles of arbitrary size instead of just the longest one.

In addition, since the Becker et al. algorithm is not able to handle side compounds, we included this possibility. Similar to Karp et al. [7] the definition of side compounds results from a predefined list with compound names (e.g. *ATP*, *NADP*, *H_2_O*, etc.) That list is editable by the user. Each compound (side and main) is treated as a node in the graph. However, in contrast to Rojdestvenski [13] we place side and main nodes simultaneously which means that as default side compounds have the same priority as the main compounds. Only during the process of cycle search, main compounds are prioritised. This default is chosen, because many examples show that the differentiation between main and side compounds is helpful at times, but often somewhat arbitrary blurring the biochemical reality. Nevertheless, it is also possible to generate a layout without any side compounds.

The list of reactions comprising the biochemical network can be submitted as an SBML [12] or a simple text file (listing all reactions separated by semicolon). It is visualised by a hyper-graph, which means that each reaction is represented by two connected dummy nodes, one is linked with all substrates and the other one with all products of this reaction.

In the following, we will describe in detail how our algorithm works. First, we will show how hierarchical and cyclic subgraphs are found and second how these subgraphs are reassembled to a whole graph.

### Identifying subgraphs

**Figure 3:**
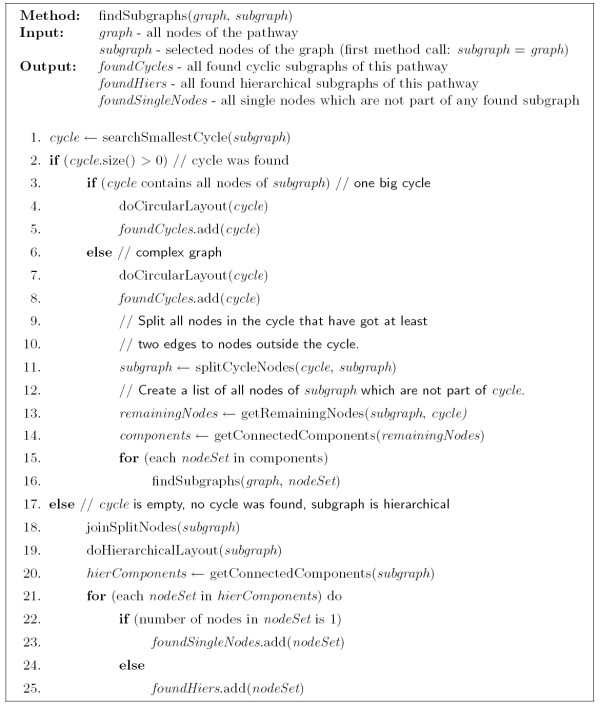
**Pseudo code of the method findSubgraphs**. This method searches for all hierarchical and cyclic subgraphs of a given pathway.

**Figure 4:**
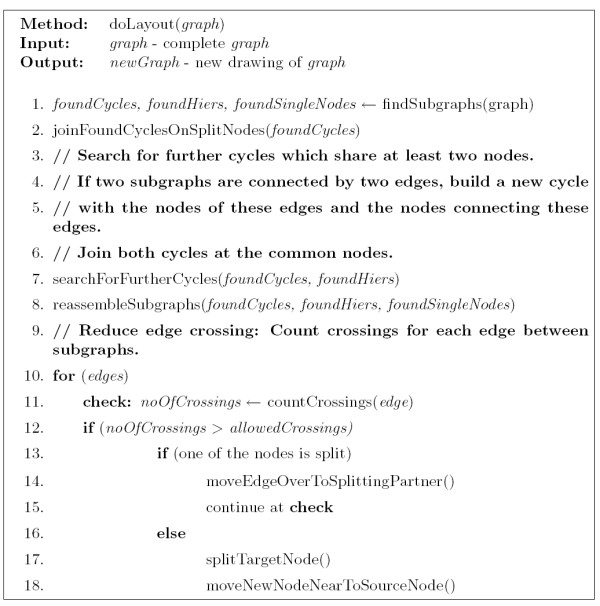
Overview of the layout process.

**Figure 5:**
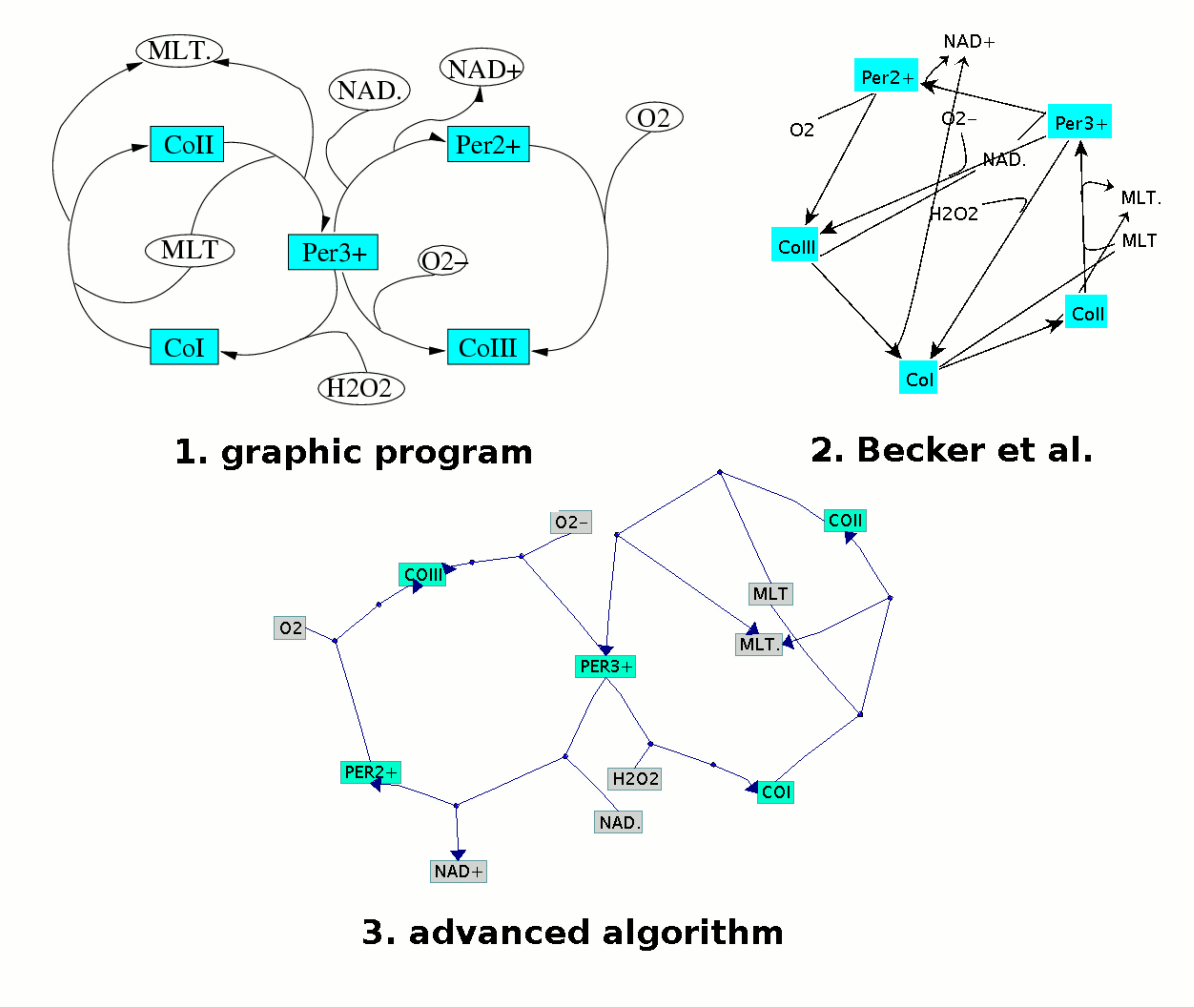
**Visualisation of a part of the PO reaction [18] (6 reactions)**. The layout of the graph that can be seen in the first picture was crafted with the aid of a graphic program by a biochemist, the second one was dynamically generated by the Becker et al. algorithm and the third one by our new algorithm.

This section describes the first part of the algorithm which identifies the cyclic and hierarchical subgraphs of a given pathway. To find joined cyclic subgraphs, nodes which are part of more than one cycle are split. The pseudo-code in Figure 3 describes the modified Becker et al. recursive method to identify circular and hierarchical subgraphs in a given pathway. The first step is to search for the smallest instead of the longest cycle (Figure 4, line 1) As explained above this procedure is chosen, since otherwise biologically relevant information might get lost, since small cycles often represent important recycling processes or short cuts in a pathway. One example is shown in Figure 5. All graphs represent a part of the Peroxidase-Oxidase reaction (PO reaction [18]). The first picture was crafted with a graphic program by a biochemist. The second one was dynamically generated with the Becker et al. algorithm. In this case, the two cycles of the first picture are not easy to depict, because of the emphasis on the longest possible cycle. However, the two cycles represent the two main recycling processes of enzyme intermediates and are therefore crucial for the reaction mechanism. These cycles are shown in the third picture in Figure 5 which is generated by our new layout algorithm.

To be identified, the smallest cycle must contain at least three compounds. However, this number is adjustable by the user, since there are of course cases where the cycle representing biologically important information is not the absolutely smallest. Therefore, if the first layout depicting the smallest cycle is not of the desired quality, the user can change it by increasing the number of compounds for the cycle search. Furthermore, the cycle must contain all the dummy nodes of each participating reaction.

In the first round of cycle searching only the main compounds and dummy nodes are used to find the smallest cycle. Then the algorithm looks for connected components. Connected components are sets of nodes which are directly or indirectly connected via edges. Thus, there exits a path between each pair of two nodes in the set. The Becker et al. algorithm looks for strongly connected components instead. In contrast to connected components, strongly connected components consider the edge direction. Thus, the above definition applies, however, the path between each pair of nodes in the respective highly connected components is only valid if the direction of the edges is always the same.

For this reason our algorithm is able to find both cycles where all edges have got the same direction (e.g. in Figure 5 the left cycle (*PER*^3+ ^- *COII - COI*) in the first picture) and also cycles where edges have got different directions (e.g in Figure 5 the right cycle (*PER*^3+ ^- *PER*^2+ ^- *COIII*) in the first picture). Finally, the breadth first search (BFS) [20] finds the smallest cycle if any exists.

If no cycle is found in the first call of the *findSubgraph *method, the whole graph will be drawn hierarchically. Otherwise, if one cycle has been found already and the method does not detect a second one with only main and dummy nodes, nodes representing side compounds are also included into the cycle search.

The algorithm keeps distinguishing between these three cases:

• No cycle found → draw the complete graph with a hierarchical layout algorithm (Figure 3, line 17)

• All nodes of the graph belong to the found cycle → draw the complete graph with a circular layout algorithm (Figure 3, line 3)

• Complex graph → draw the found cycle with a circular layout algorithm and separate the remaining nodes of the pathway into further cyclic and hierarchical subgraphs (Figure 3, line 6)

In the first case the Becker et al. algorithm uses a standard hierarchical layout algorithm. We improved this standard algorithm by separating the placement of the nodes into two steps. First the main and dummy nodes are placed by the standard algorithm. Second the side compounds are split to create as many nodes as occurrences in reactions exist. Hence, every node has got only one edge. These nodes are positioned one layer above or under the other end point of the edge according to the direction (top to down).

In the second case the whole graph consists of one cycle and all nodes are therefore positioned by a standard circular layout algorithm.

**Figure 6:**
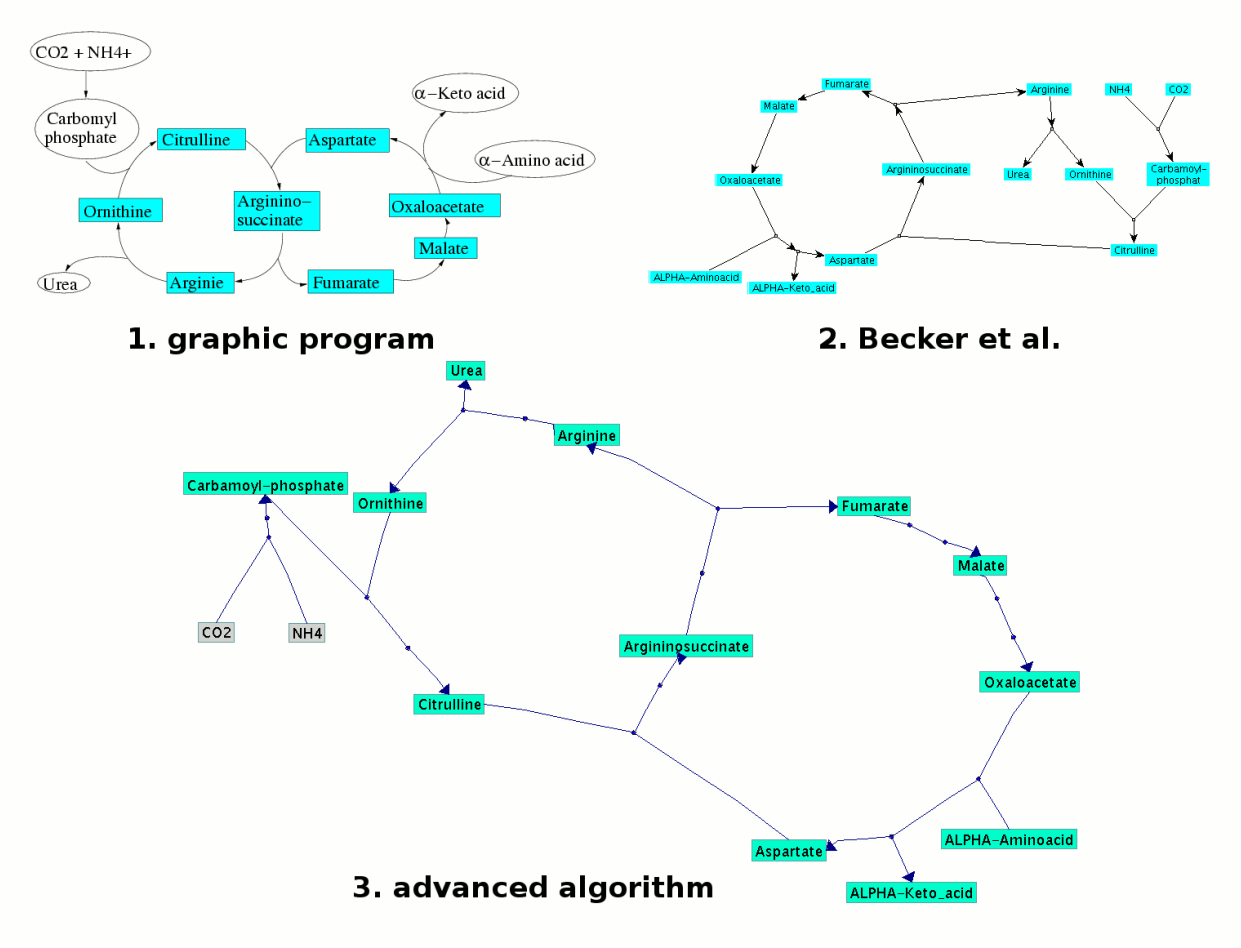
**Visualisation of the urea cycle and parts of the citrate cycle**. The first picture was made manually, the second one was dynamically generated by the Becker et al. algorithm and the third one by our new layout algorithm. All visualisations look similar but the two cycles in the first and third picture are not well represented in the middle one.

In the complex graph case the graph consists of various circular and hierarchical subgraphs. Since in all existing layout algorithms each node is part of exactly one subgraph, these algorithms are not able to find cycles which share nodes. Therefore, we added the possibility to split (Figure 3, line 11) and join nodes (Figure 3, line 18 and Figure 4, line 2). Figure 6 shows the urea cycle and a part of the citrate cycle crafted with a graphical program by a biochemist. The Becker et al. algorithm finds the urea cycle and considers the unshared parts of the citrate cycle as hierarchical subgraph. In contrast to this picture our new algorithm finds two cycles joined at **argininosuccinate**.

**Figure 7:**
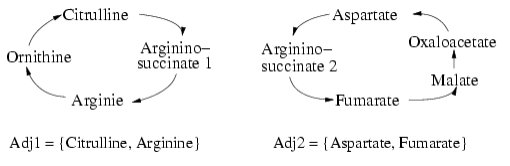
**Splitting nodes**. This figure shows an example how a node, in this case **argininosuccinate **is split into two nodes. The adjacency list (Adjx) shows which edge belongs now to which node.

This result is achieved by splitting nodes in found cycles which could also be part of another cycle (Figure 3, line 11). These nodes must represent compounds and must have at least four edges, two edges to nodes in the found cycle and two edges to nodes which are not part of this cycle. Dummy nodes are not allowed to split. Each node *n *is split into *n*_1 _(node with all edges connecting nodes in the found cycle) and *n*_2 _(node with all remaining edges), see also Figure 7. In this way, several cycles representing biochemical reaction cycles that share a compound can be found and represented accordingly.

When no more cycles are found, the remaining nodes are regarded as hierarchical. Split nodes are joined and the subgraph is inspected to find connected components before the improved hierarchical layout algorithm places the nodes of this subgraph (Figure 3, line 18). Each connected component is considered as one hierarchical subgraph but components with only one node are saved in an extra set (Figure 3, lines 20–24) and are placed separately.

### Building the complete graph

In this section the subgraphs are reassembled to a complete graph by

• Joining split nodes if possible (= joining cycles at one node, Figure 4, line 2).

• Search for further cycles in the found subgraphs (Figure 4, lines 3–7).

• Reassembling of the found subgraphs to a complete graph using a force-directed layout algorithm (Figure 4, line 8).

• Reducing edge crossings between subgraphs (Figure 4, lines 9–18).

These processes are described in detail in the following paragraphs.

For the joining of split nodes present in cycles, the algorithm tries to join main nodes with priority over side nodes. Therefore, cycles with split main nodes will be joined before cycles with split side nodes. Joining two nodes means that two cycles are rotated and moved together at these nodes. One node will be deleted from the graph and all its edges will be shifted to the other one (see an example in Figure 5 (*PER*^3+^)). Only two cycles are allowed to be joined at the same node because more than two cycles would cause edge crossings.

For the detection of further cycles, the algorithm searches for subgraphs which are connected by at least two edges with another subgraph. These edges must have got different source and target nodes. If such edges exists, the algorithm will determine the shortest path between the nodes of these two edges in both subgraphs. The found nodes of this path are used to build a new cycle. This new cycle must also correspond to the above explained definition of a valid cycle and is then drawn accordingly. The new cycle and the already existing one will be joined at the common nodes. See an example in Figure 6, the two cycles are joined at **argininosuccinate **and the four dummy nodes of the two reactions in which **argininosuccinate **participates.

In contrast to the Becker et al. algorithm the final reassembling step only starts after all subgraphs are found and split nodes are joined. The force-directed method takes the cycle with the maximal number of edges to other subgraphs as central subgraph and all remaining subgraphs and single nodes as input. The force-directed algorithm places all subgraphs around the central cycle to build the complete graph. Finally, to reduce edge crossings, all edges between found subgraphs are checked. The list of these edges is sorted by their length in descending order because typically the longer the edge the higher the number of edge crossings. Starting from the longest edge the number of edge crossings is counted for each edge of this list and nodes are split to reduce the number of crossings. This number of allowed edge crossings can also be changed by the user.

Before splitting a node the algorithm checks whether one node of this edge was the result of another splitting operation and checks whether its splitting partner could take over this edge with at most two edge crossings. If this is not possible, the target node of the examined edge is split and a new node is created that has got only this edge and is placed near to the source node. Nodes with only one edge are moved directly near to the other edge endpoint. To generate a planar graph the number of allowed edge crossings can be set to zero but that also increases the number of split nodes.

In addition, the placement of labels is automatically done by the used layout algorithms of the YFiles package.

## Discussion

We have presented our new dynamical layout algorithm for metabolic pathways. One of the main differences to existing algorithms is the emphasis on finding small cycles. This results in a biochemically meaningful representation in many cases, since cycles in biochemical networks often stand for important processes like recycling of intermediates, energy or electron carrier producing or futile cycles. Therefore, biochemists are used to seeing these processes as graphical cycles and our algorithm takes care of this convention. For those cases where the smallest meaningful cycle does not match the default settings, these can be easily adjusted.

Our algorithm is able to handle linear, cyclic and complex metabolic pathways considering main and side compounds. A complex pathway consists of diverse hierarchical and cyclic subgraphs. Nodes are split and joined to improve the detection of these subgraphs and to minimise edge crossings. Finally in many cases the drawing reflects the biological context better than previous approaches, e.g. cycles which share at least one node can be found and represented.

Therefore, our algorithm satisfies the following constraints:

1. *Considering further biological conventions: *By identifying cycles which share at least one node and by splitting nodes, pathways are drawn more accurately and more similarly to the visualisations biologists are used to (e.g. Figure 6).

2. *Unequivocal distinction between substrates and products: *Each reaction is represented by two connected dummy nodes which allows an exact distinction between products and substrates in each reaction.

3. *Complexity reduction: *The complexity of pathways is reduced by splitting higher connected nodes (see also above). Thereby it is possible to untangle nets of many edges to one node, see KEGG [21] pyruvate metabolism (pathway no. 00630, e.g. **Glyoxylate**).

4. *Edge crossing minimisation: *The edge crossings are minimised in the second splitting phase when one node of an edge with more than two edge intersections is split.

5. *Edge length reduction: *Both dummy nodes of one reaction must be part of the same subgraph which minimises the distance between two dummy nodes. As mentioned above, compounds are split and placed near to each other to minimise edge crossing which also reduces the edge length. These methods also fulfil the constraint that compounds of the same reaction should be placed near to each other.

Since our algorithm is based on the Becker et al. algorithm, it calculates similar results for the examples optimally represented in the Becker at al. publication. These examples and several other pathways from the databases KEGG [21], BioCyc [22] and PathDB [23] drawn by the new dynamical layout algorithm can be found at [2]. In addition, since we want to support simulation and modelling tools, we used SBML files describing models of biochemical networks from the SBML model repository ([24]) and the model database ([25]).

The Mendes et al. algorithm (PathDB) can only be used by a general user in the context of the respective database and therefore just with the pathways stored in those. For this reason we restricted ourselves to these pathways to make a comparison.

In the case of the Karp et al. (BioCyc) algorithm which normally is also used in the context of a database, we were able to compare the algorithm in an isolated manner, since it was generously supplied by Karp and coworkers. The isolated algorithm performed well on small to medium sized samples, however, faced some problems w.r.t. edge crossings when considering larger or higher connected pathways (data not shown). In addition to the information on the individual reactions, the isolated algorithm also uses information about the order of the reaction events which is absent from model files, e.g. SBML files. However, this probably could be easily circumvented by a preprocessing step of the respective SBML file if wanted.

The Mendes et al. algorithm and to a lesser extend the Karp et al. algorithm usually use additional information about the considered pathway from their database, e.g. the order of reaction events as pointed out above, to simplify the layout process.

Since such information is not available to a simulation/modelling tool, our algorithm relies solely on a list of reactions of the pathway and optionally a predefined list of side compounds. The existing layout algorithms for metabolic pathways treat the side and main compounds of a pathway differently from our algorithm. They all treat the side compounds as labels, which results in the labels overlapping in complex pathways. Although side compounds are part of the graph in our algorithm the algorithm produces similar results compared to the existing algorithms in those cases where the latter produce good results and solves the overlapping problem in the more complex cases.

**Figure 8:**
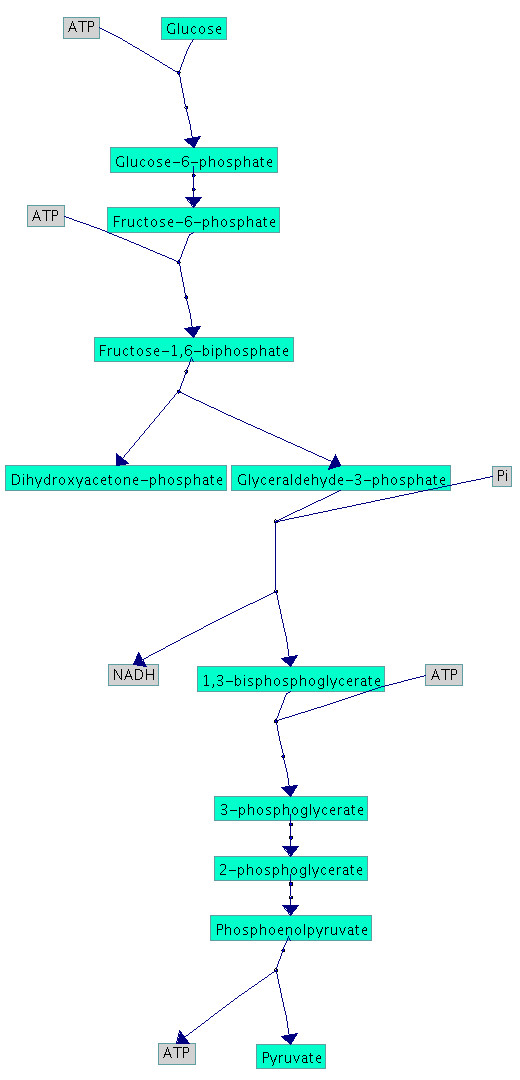
**Visualisation of glycolysis**. In this picture *ATP*, *Pi *and *NADH *are considered as side compounds which shows the known hierarchical structure of the glycolysis.

**Figure 9:**
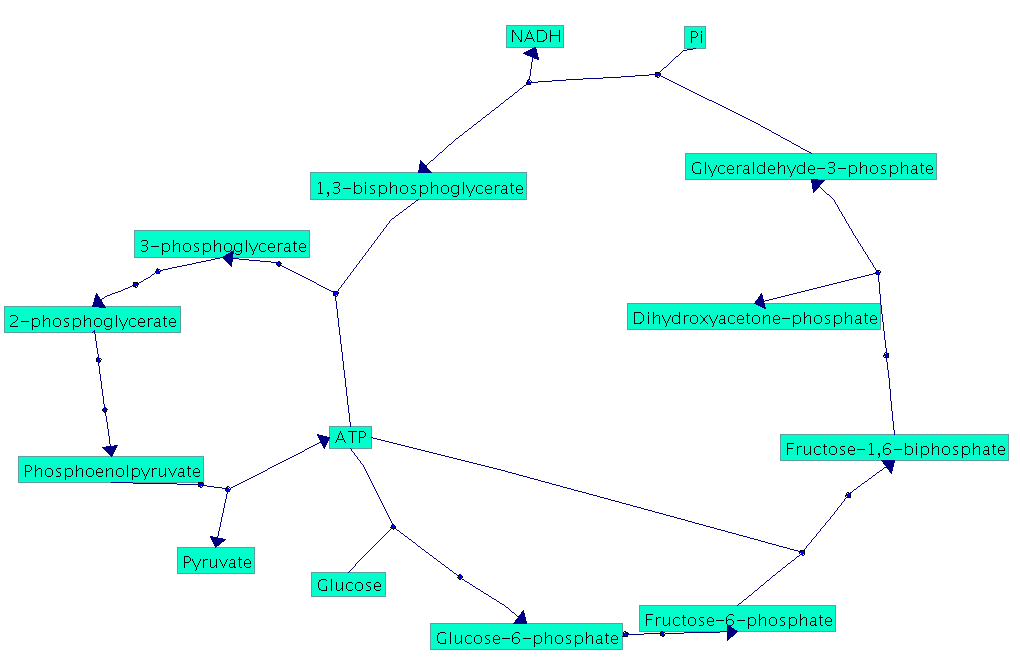
**Visualisation of glycolysis**. In contrast to Figure 6 all compounds are treated as main compounds and the result consists of two cycles which are joined at *ATP*. This example shows the importance of the choice of the side compound and demonstrates its influence on the topology of a pathway.

The user defined list of side compounds naturally influences the treatment of the nodes during the layout process. By editing this list the user should keep in mind that different side compound lists lead to different drawings of the same pathway, see Figure 8 and 9. Since some parts of the algorithm rely on stochastic methods, the same pathway could be represented with different layouts. For example, the force-directed layout algorithm could cause different assemblings of the subgraphs. In addition the breadth first search of the cycle finding process checks the nodes according to their number of edges in descending order. If there are different nodes with the same number, the order is chosen randomly which means a varying order of nodes could lead to different cycles.

To finish, some words about the complexity of the algorithm. The bottleneck is the cycle search. In the worst case, a breadth first tree for every node has to be calculated when no cycle of the given definition exists. In this case, the complexity of this process is *N*^3 ^(N = number of nodes). However, if a cycle exists the complexity is much lower leading to faster results.

In the near future we want to integrate standard layout algorithms independent from YFiles in order to be able to e.g. change straight lines in cycles into curves and we want to integrate enzymes and regulators as nodes.

## Authors' contributions

KW carried out the development and implementation of the algorithm and participated in discussions and writing.

UK initiated the project and participated in discussions and writing.
